# Surgical Treatment of Enchondromas of the Hand: Our Experience in Curettage Only and Early Mobilization

**DOI:** 10.3390/diseases13030084

**Published:** 2025-03-16

**Authors:** Silvia Pietramala, Giuseppe Rovere, Camilla Ravaioli, Ciro Mignano, Amarildo Smakaj, Andrea Fidanza, Pasquale Farsetti, Lorenzo Rocchi, Camillo Fulchignoni

**Affiliations:** 1 Department of Orthopaedics and Traumatology, Fondazione Policlinico A. Gemelli IRCCS-Università Cattolica del Sacro Cuore, 00168 Rome, Italy; camilla.ravaioli01@icatt.it (C.R.); ciro.mignano01@icatt.it (C.M.); 2 Department of Orthopaedics and Traumatology, Catholic University of Sacred Heart, Roma (IT) Largo Francesco Vito 1, 00168 Rome, Italy; giuseppe.rovere02@icatt.it (G.R.); amarildo.smakaj@gmail.com (A.S.); lorenzo.rocchi@policlinicogemelli.it (L.R.); camillo.fulchignoni2@guest.policlinicogemelli.it (C.F.); 3 Department of Clinical Science and Translational Medicine, Section of Orthopaedics and Traumatology, University of Rome “Tor Vergata”, 00133 Rome, Italy; 4 Unit of Orthopaedics, Department of Life, Health and Environmental Sciences, University of L’Aquila (IT), 67100 L’Aquila, Italy; andrea.fidanza@univaq.it; 5 Orthopaedics and Hand Surgery Unit, IRCSS Policlinico Gemelli, 00168 Rome, Italy

**Keywords:** enchondroma, hand, curettage, treatment

## Abstract

(1) Background: Enchondroma is one of the most common primary tumors of the hand. Usually asymptomatic, it can present with pain, deformity, and sometimes pathologic fractures. Surgical treatment is advised in these cases. Curettage is the basic treatment, but there is no consensus in the literature regarding post-void filling. The aim of our study is to present simple curettage and early mobilization as a safe and effective treatment. (2) Methods: We retrospectively analyzed patients treated at our center between 2020 and 2024. Each patient was treated with simple curettage and early mobilization. We collected demographic data and follow-up data. (3) Conclusions: We recorded no complications in our cohort, pointing out that our method is safe and reliable without any kind of immobilization. Bone grafts and other methods such as cement are good options but should be considered in specific cases.

## 1. Introduction

Among the primary tumors of the hand, enchondroma is the most common one [1]. It is a benign, slow-growing, hyaline cartilage tumor that is most often located in the small tubular bones [2]. The origin is from abnormal chondromatous tissue near the central part of the involved epiphyseal plates [3]. The most affected population is in the third and fourth decades of life [3,4]. Enchondromas represent about 25% of all benign tumors and 12% of bone tumors of cartilaginous origin, excluding the hand. The ratio changes drastically when referring to the hand, as enchondromas represent about 86% of cartilaginous tumors in this area [5]. Enchondromas have a predilection for the ulnar-side tubular bones of the hand and arise most frequently in the phalanges, especially the proximal ones (48.9%), and metacarpals [4,6,7].

In most cases, they occur as solitary lesions, but they can also show up as multiples in Ollier disease and Maffucci syndrome [1,2]. In these enchondromatosis syndromes, malignant degeneration in chondrosarcomas is more common [3].

They are often asymptomatic (>50% of cases) and are diagnosed incidentally after radiographs taken for other conditions. Clinically they can present with pain, deformity, swelling, finger hindrance, and pathological fracture. Despite their benign behavior, in fact, these lesions can compromise the structural integrity of the bone [6,8].

Another clinical presentation of enchondromas is within a history of trauma. The presence of a lytic lesion inside the bone creates a weak area that is more vulnerable to trauma, ending in pathological fractures.

**Figure 1:**
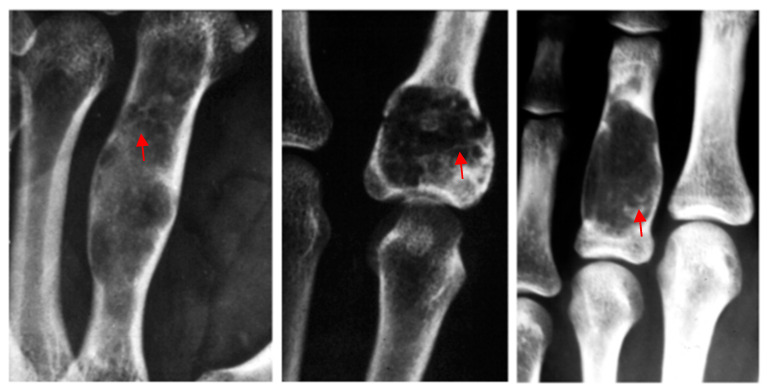
Radiologically, the typical presentation of an enchondroma is an intramedullary well-defined lytic lesion located in the middle of the phalanx with a calcified matrix (red arrow).

Radiologically, the typical presentation of an enchondroma is an intramedullary well-defined lytic lesion located in the middle of the phalanx with a calcified matrix [1] (Figure 1). No periosteal reaction is observed, but a cortical expansion can be present, especially in short bones. A radiological variance of enchondromas is *eccondroma*, which develops by a periosteal expansion.

Additional imaging is required to perform differential diagnosis and surgery planning. While a CT-scan is useful to enhance the margin’s lobulations, an MRI shows a hyperintense and shapely defined lesion with internal foci of low signal of “rings and arcs” in T2 [9]. As for the differential diagnosis, it is important to distinguish enchondroma from low-grade chondrosarcoma before surgery. The main feature that differentiates an enchondroma from a low-grade chondrosarcoma in an X-RAY is the presence of deep endosteal scalloping involving more than two-thirds of the cortical thickness. On MRI, chondrosarcoma shows uneven gadolinium enhancement in T2; however, scintigraphy is not useful as increased uptake is present in enchondroma as well [10]. As also defined during the last Birmingham Orthopaedic Oncology Meeting, several studies have described predictive classification systems to differentiate atypical cartilage tumors from chondrosarcoma, based on MRI with high sensitivity and specificity, though these are not yet in routine practice [11]. Other differential diagnoses are epidermoid cysts, other types of cartilaginous tumors, and giant cell tumors [12].

Macroscopically, enchondromas show a lobular cartilaginous aspect with a characteristic white/bluish color. The texture can be thick or soft, and the margins are usually clear but indented [13]. The final diagnosis relies on histologic examination. Microscopically, they are usually composed of lobules of cartilaginous tissue divided by septa of connective tissue, but, in the hands and feet, the features might change, similarly to low-grade chondrosarcoma or atypical cartilaginous tumors. The most important and reliable histopathological criteria for distinguishing between atypical cartilaginous tumors and enchondromas are the presence of a myxoid tumor matrix, infiltrative growth, and endosteal scalloping [14].

In the case of enchondromas discovered incidentally, showing no symptoms even in the presence of deformity, observation can be a valid option. Muller et al. [15] reported a case series of 73 patients treated with regular radiological follow-up. The main reason behind conservative treatment lies in the possible complications of surgery including long immobilization periods and a certain degree of loss of motion.

Commonly, symptomatic enchondromas and pathologic fractures are treated surgically. Despite this tumor’s frequent occurrence in the hand, its treatment lacks standardized protocols. The most used treatment is curettage [1,7]. Post-void filling is a topic of open debate as the literature describes different options such as autogenous and allogeneic bones, synthetic bone, and other substitutes such as hydroxyapatite and CPC (calcium phosphate bone cement) [16,17].

These alternatives, especially those involving a donor site, could lead to different complications including higher infection rates, long immobilization periods, and loss of function. For these reasons, conservative treatment is often considered as a valid option. Our thorough literature search pointed out how little consideration is given to simple curettage only as only a few authors described their experience [3,18,19,20,21] and, moreover, the majority of case series were dated before the 2000s. We believe that the enthusiasm behind bone grafts and more innovative substances has left this reliable technique behind. Our approach not only supports avoiding post-void filling but presents it as a safe technique that does not need post-operative immobilization. With these premises, the aim of this study is to prove the effectiveness of the surgical treatment with curettage only combined with early mobilization as a reliable option to reduce post-operative stiffness without increasing the risk of recurrence and complications.

## 2. Materials and Methods

This study was conducted in accordance with the Declaration of Helsinki.

### 2.1. Patient Selection

All the patients who underwent curettage surgery for cartilaginous tumors of the phalanx or metacarpal bone at our institution between January 2020 and October 2024 were selected. The inclusion criteria were as follows: (1) final diagnosis of enchondroma or benign cartilaginous tumors located at the phalanx and metacarpal bone; (2) surgical curettage without void filling; (3) at least 12 months follow-up post-operatively.

The exclusion criteria were as follows: (1) diagnosis not confirmed after histologic examination; (2) patients lost at follow-up; (3) patients who underwent additional therapies (e.g., radio frequencies); (4) patients with acute fractures.

### 2.2. Data Collection

The data were extracted from our institution’s system by searching the following words in our database: “enchondroma” AND “hand” AND “phalanx” OR “metacarpal”. We collected demographic data, including age, gender, comorbidities as well as clinical presentation, symptoms referred by the patients, and imaging provided. A history of fractures was recorded. If applicable, the time of splinting was also registered. Surgical complications, intended as any deviation from the normal post-operative course, were recorded during the first two planned post-operative check-ups. Recurrence was defined after at least 12 months follow-up.

### 2.3. Surgical Technique

**Figure 2:**
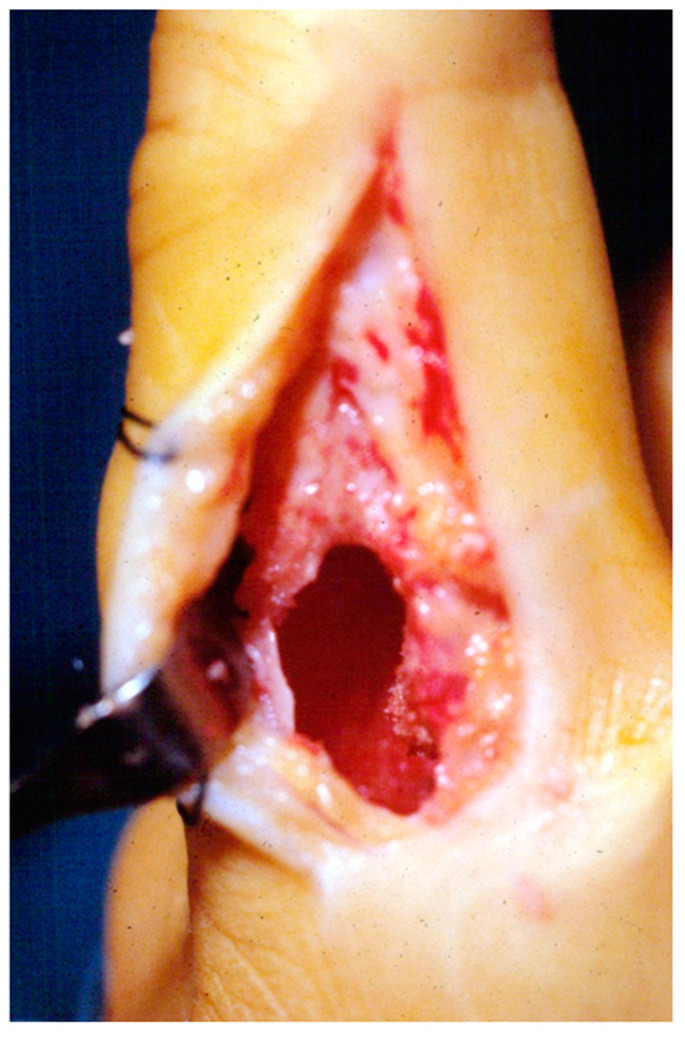
Surgical access shown on the lateral aspect of a proximal phalanx. The cortical surface is opened by the use of a surgical awl and curettage is performed with a curette, to the point of breaking the tumor cavities.

All the surgeries were performed under brachial plexus anesthesia with a tourniquet. In the case of enchondromas of the phalanx, a longitudinal lateral incision was made. Under fluoroscopy, the entry point into the bone was identified and realized by a surgical awl or a small-sized osteotome. At this point, a *locus minorisresistentiae* must be identified (Figure 2).

**Figure 3:**
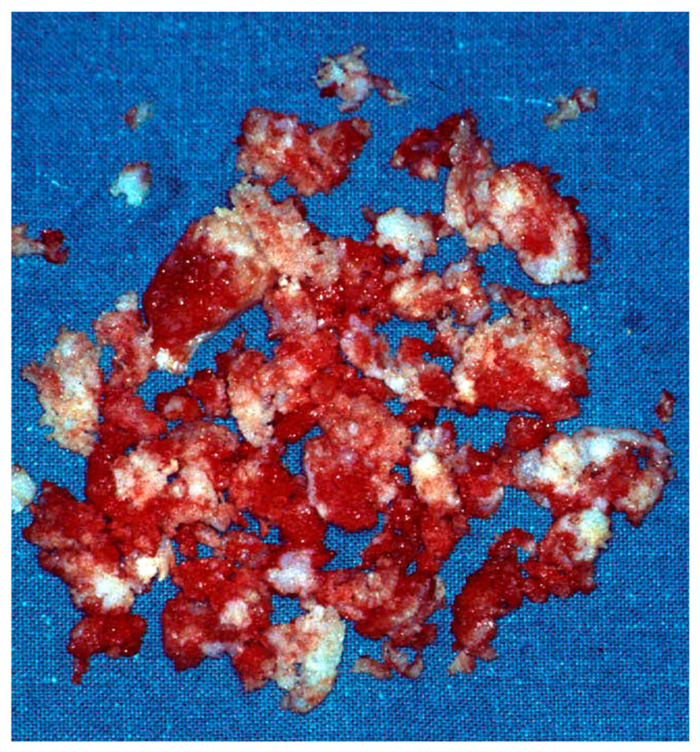
Macroscopical appearance of hand enchondroma. It shows a lobular cartilaginous aspect with white/bluish color. The margins are usually clear but indented.

By the use of a curette, the chondromatous material was completely excised until normal bone was found [Figure 3].

After the whole cavity is emptied, the walls of the cavity must be broken, reaching the intramedullary canal. In this way, new osteoblastic chips can penetrate the former cavity to form new bone and proceed with the healing process. This technique was initially described by Fanfani in 1986 [19].

### 2.4. Post-Operative Care

After surgery, no immobilization was placed. A simple bandage on the single digit or, especially in the case of extreme cortical thinning in phalanx enchondromas, a syndactyly was placed. The patients were left free to move, avoiding carrying weight and practicing sports at risk of injury. Stitches were removed after 15–20 days, and no intermediate dressing change was scheduled. In case of stiffness, the patients were educated to perform self-assisted mobility at home with no limits of ROM. Physical therapy was advised only under the patient’s request. If an intraoperative fracture was recorded, a dorsal Zimmer splint was applied blocking one joint only in a resting position for 30 days. An X-RAY was requested at 1 year, followed by a CT-scan in the case of a doubtful result. 

## 3. Results

From January 2020 to January 2024, we collected 50 patients with a diagnosis of enchondroma. Of these, 10 patients were excluded as they presented with an acute pathological fracture, while 8 were excluded as they were treated with a bone graft. All the lesions were confirmed as enchondroma by histologic examination. Finally, 32 patients with a diagnosis of enchondroma treated with simple curettage and early mobilization were considered eligible (Table 1).

**Figure 4:**
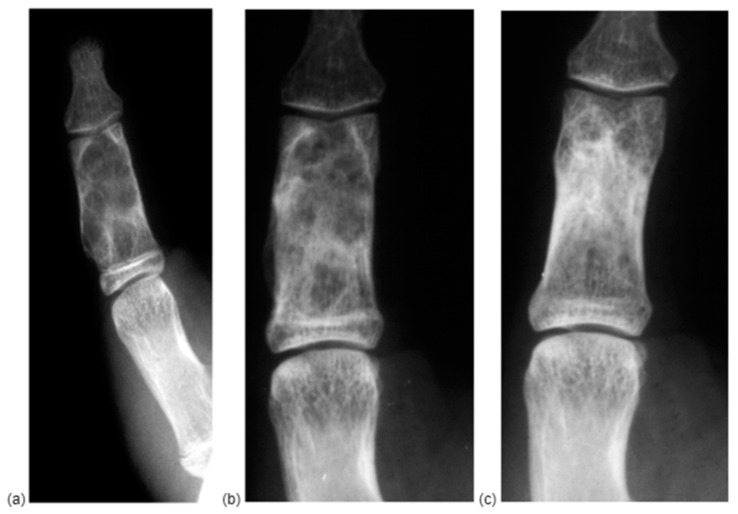
X-RAY of a P2D2 enchondroma at first diagnosis (**a**), at 2 months (**b**), showing a fast reconstruction of the cancellous bone with a slower remodeling of the normal morphology of the bone, which was obtained at 12 months (**c**).

Our cohort included 20 females (62.5%) and 12 males (37.5%). The mean age was 44.06 years old (range 10–87). The most affected digit was the fourth (37.5%), followed by the second (28.12%). Only seven cases reported a metacarpal enchondroma. While 34.37% of patients reported no symptoms and the diagnosis was occasional after a blunt hand trauma, the most common symptom was pain accompanied with local deformity or a lump. An X-ray was requested in all the cases, while a CT-scan was available only in five cases, and, in each of them, a history of fracture was recorded. At the 2-month follow-up, initial cancellous bone reconstruction already showed in 60% of cases, while complete healing was observed in 100% of cases at 12 months. Immobilization was required in one patient only as after complete enchondroma excision, extreme cortical thinning was observed. The mean follow-up was 23.3 days, but each patient was evaluated through X-RAY at 2 months and 1 year. In total, 60% of patients evaluated at 2 months showed initial cancellous bone remodeling, while healing was observed in 100% of cases at 1 year, and no recurrence was found (Figure 4).

The average time to return to work was 20 days. We registered only one case of recurrence: patient number 12, who was taken in charge at his third recurrence and that was eventually addressed by ray amputation, expressly asked for by the patient, due to persistent pain and deformity. Other complications were hypertrophic scarring and PIP stiffness, which resolved after physical therapy.

## 4. Discussion

The utility of different void-augmentation methods following curettage in the surgical management of hand enchondroma continues to be a subject of debate [6]. While some recommend the use of grafts or substitutes, their role remains uncertain. This study seeks to demonstrate that performing curettage only, combined with early mobilization, is both a safe and an effective strategy. Our clinical findings support this approach, showing outcomes comparable to those achieved with additional interventions, without the additional risks of augmentation procedures/the added complexity of augmentation procedures such as the increased risk of infection and post-operative stiffness. A surgical approach must be performed with a triple purpose: eradicating the enchondroma, minimizing the risk of local recurrence, and resolving the clinical picture for which it is responsible. 

The most reported surgical technique in the literature is to fill the bone defect with autologous bone chips, usually taken from the iliac crest [20]. However, bone harvesting from the iliac crest requires general anesthesia and prolonged surgical time and can sometimes cause continued pain/discomfort at the donor site in 7% of patients [6,21]. To avoid the necessity of harvesting cancellous bone from a separate surgical site, the defect can be filled with bone substitutes, such as hydroxyapatite. However, this material has been known to cause soft-tissue irritation, leading to pain [22]. Other authors have replaced hydroxyapatite with another allograft such as CPC (calcium phosphate bone cement), which does not induce inflammatory reactions in soft tissue and gives immediate mechanical stability [23], despite being too expensive. Extended preparation time, administration challenges, toxicity, setting duration, and cost are additional disadvantages that should be considered when planning to use CPC or other types of bone cement to fill voids. Other possible disadvantages are foreign body reaction, delayed integration, insufficient defect consolidation [24]. 

Concerning the consolidation period, autogenous bone grafting is the fastest, although allogeneic bone grafting shows comparable results. A study reported that, in a sample of 76 patients with a 15-year follow-up, autografts took an average of 38 days for consolidation, while allografts took an average of 51 days [25]; meanwhile, it is important to consider that early movement is crucial to prevent finger contracture in the treatment of hand enchondroma. When using a bone graft, an immobilization, as a palmar splint or a metal finger splint, is usually carried out for about 4.5 weeks (with a maximum of 6 weeks) until radiological union is observed [26]. Bone regeneration may occur naturally without the need for additional interventions such as bone grafting or cement injection after curettage. Other authors like Morii et al. [24] compared the bone formation period between patients treated with simple curettage and those who received hydroxyapatite injection, finding no significant difference between the two groups. Similarly, Schaller and Baer reported that there was no significant difference in bone density or functional outcomes between 16 patients who did not receive a bone graft and 8 patients who did [25]. In our series, we observed only one recurrence, and, at 12 months, bone regeneration was observed in all the other cases. It must be underlined that it is crucial to break enchondroma cavities to let osteoblasts penetrate the intramedullary canal to promote new bone formation. The main difference between our series and those described in the literature is the period of immobilization. Bachoura et al. [3] described an average immobilization period of 2 weeks for distal and middle phalanx enchondroma and a 6-week splint in the case of proximal phalanx or metacarpal involvement.

We purposely excluded pathological fractures as the treatment is different, especially regarding the immobilization period. As reported in a review of 40 cases [17], the group with the most satisfactory outcome was the one in which surgery was avoided. By now, observation is the treatment of choice in the case of fractures related to enchondromas, and this is also our treatment of choice. In fact, it is not infrequent to observe tumor reabsorption along with the fracture healing. For this reason, we believe that enchondroma must not be treated during an episode of fracture. As also reported by Ablove [26] and colleagues, there is a higher complication rate with immediate surgical treatment, consisting in loss of motion and fracture displacement. 

Curettage only and early mobilization is a valid, low-cost and low risk of complication treatment option for most hand enchondromas [27], but we recognize that our study has some limitations. This type of tumor is rare, and so this did not allow us to operate on a larger population. An additional limitation is the retrospective approach of our study, which made it difficult to standardize post-operative follow-up procedures for each patient. A control group of patients treated with bone grafts could be useful to compare results especially in terms of functional outcomes. Although we sometimes use bone grafts, we believe that this technique must be reserved for precise cases such as extreme cortical thinning or intraoperative fracture.

## Figures and Tables

**Table 1:** Cohort characteristics.

ID	Gender	Age	Location	Symptoms	Imaging	Fracture	Immobilization	FUP	RTW
1	F	27	P1D4	Pain/deformity	X-RAY	yes	no	12	30
2	F	32	P2D4	Pain	X-RAY	no	no	12	20
3	F	51	P2D2	Pain/deformity	X-RAY/MRI	no	no	12	20
4	M	67	5MC	Pain, stiffness	X-RAY/CT	yes	no	12	/
5	M	25	PF1D2	Stiff PIP flexion	X-RAY	no	no	20	20
6	F	72	PF1D4	Stiff PIP	X-RAY	yes	no	22	/
7	F	56	3MC	No	X-RAY	no	no	36	20
8	M	34	4MC	No	X-RAY	yes	no	36	40
9	F	60	P2D2	No	X-RAY	no	yes	38	20
10	F	49	P1D4	No	X-RAY	yes	no	40	20
11	F	10	P2D2	Swelling	X-RAY	no	no	24	/
12	M	87	P1D2	Swelling	X-RAY/CT	yes	no	30	/
13	F	45	P2D3	No	X-RAY	no	no	25	20
14	F	48	3MC	Swelling	X-RAY	yes	no	30	25
15	F	26	P1D5	No	X-RAY	no	no	12	20
16	M	45	P1D4	Pain/deformity	X-RAY	no	no	18	23
17	M	56	P2D4	Pain, stiffness	X-RAY	yes	no	22	15
18	F	28	P2D2	Stiff PIP flexion	X-RAY	yes	no	31	30
19	F	42	5MC	No	X-RAY	no	no	36	20
20	F	54	P1D4	No	X-RAY/CT	yes	no	40	18
21	M	61	P2D4	Swelling	X-RAY	yes	no	12	15
22	M	19	P1D2	Swelling	X-RAY	yes	no	24	/
23	M	23	P2D3	No	X-RAY/CT	yes	no	28	20
24	F	21	P1D2	Swelling	X-RAY	no	no	24	25
25	M	42	P1D5	No	X-RAY	no	no	24	20
26	F	27	P1D4	Pain/deformity	X-RAY	yes	no	12	30
27	F	32	P2D4	Pain	X-RAY	no	no	12	20
28	F	51	P2D2	Pain/deformity	X-RAY/MRI	no	no	12	20
29	M	67	5MC	Pain, stiffness	X-RAY/CT	yes	no	12	/
30	M	25	PF1D2	Stiff PIP flexion	X-RAY	no	no	20	20
31	F	72	PF1D4	Stiff PIP	X-RAY	yes	no	22	/
32	F	56	3MC	No	X-RAY	no	no	36	20

P = phalanx, D = digit, MC = metacarpal, PIP = proximal interphalangeal, CT = computer tomography, MRI = magnetic resonance imaging, FUP = follow-up, RTW = return to work.

## Data Availability

Data are available in the main text, and supplementary data are available under request to the corresponding author.
